# SD-Net: joint surgical gesture recognition and skill assessment

**DOI:** 10.1007/s11548-021-02495-x

**Published:** 2021-10-16

**Authors:** Jinglu Zhang, Yinyu Nie, Yao Lyu, Xiaosong Yang, Jian Chang, Jian Jun Zhang

**Affiliations:** 1 National Centre for Computer Animation, Bournemouth University, Bournemouth, UK; 2 Technical University of Munich, Munich, Germany

**Keywords:** Surgical gesture recognition, Temporal convolutional network, Self-attention, Surgical skill assessment

## Abstract

**Purpose:**

Surgical gesture recognition has been an essential task for providing intraoperative context-aware assistance and scheduling clinical resources. However, previous methods present limitations in catching long-range temporal information, and many of them require additional sensors. To address these challenges, we propose a symmetric dilated network, namely **SD-Net**, to jointly recognize surgical gestures and assess surgical skill levels only using RGB surgical video sequences.

**Methods:**

We utilize symmetric 1D temporal dilated convolution layers to hierarchically capture gesture clues under different receptive fields such that features in different time span can be aggregated. In addition, a self-attention network is bridged in the middle to calculate the global frame-to-frame relativity.

**Results:**

We evaluate our method on a robotic suturing task from the JIGSAWS dataset. The gesture recognition task largely outperforms the state of the arts on the frame-wise accuracy up to $\sim$ **6** points and the F1@50 score $\sim$ **8** points. We also keep the 100% predicted accuracy for the skill assessment task using LOSO validation scheme.

**Conclusion:**

The results indicate that our architecture is able to obtain representative surgical video features by extensively considering the spatial, temporal and relational context from raw video input. Furthermore, the better performance in multi-task learning implies that surgical skill assessment has a complementary effects to gesture recognition task.

## Introduction

There has been a growing interest in building context-aware system (CAS) utilizing available information inside the operation room (OR) to provide clinicians with contextual support. It allows various applications through the whole patient care pathway, such as clinical resources scheduling and report generation [15]. Among related techniques of context-aware assistance, automatic surgical gesture recognition is an essential component to understand the surgical video content. However, the operation environment is considerably complicated. For one, surgical activities share similar environment due to the similar appearance, color and texture of human anatomic structure. For another, surgical process is specific to the medical condition, the surgeon and the patient, such that the process varies significantly from one to another. Accordingly, it is quite challenging to segment surgical gestures with low inter-class variance and high intra-class variance from long and untrimmed videos.

Surgical gesture recognition aims to classify the fine-grained surgical actions with their corresponding boundaries. Some of the prior studies apply hidden Markov model (HMM) and its variants [10, 19] and conditional random fields (CRFs) [16] to model the latent state transition of successive surgical actions by transition probability. Although the results are interpretative and promising for these probabilistic graphic models, they only focus on few neighbor frames, and it requires dense kinematic annotations, which is not always available during a surgery. Other studies manually design multiple features (intensity, color, motion, etc.) and use machine learning models such as support vector machine (SVM) [20] to segment and predict surgical activities. Nonetheless, handcrafted features selection is an empirical process. Some latent and significant features could be overlooked during the feature extraction stage.

Recently, various deep learning techniques have been proposed to capture the video temporal information. Recurrent neural network (RNN), particularly the long short-term memory (LSTM) network [3, 18], is able to conserve the critical contextual memory while drop the unrelated information by its gate mechanism. But the vanishing gradient during back-propagation [17] limits its ability of capturing long-range temporal features. Liu et al. [14] use deep reinforcement learning algorithm to model the task as a sequential decision-making process and reduce the over-segmentation error. Every time step, the agent looks through the video sequence or kinematic information from the beginning and gradually learns a strategical policy to classify the frame based on the reward. Temporal convolution networks (TCNs) [4, 12] are then proposed to hierarchically aggregate video dynamics by 1-D convolutions and de-convolutions with different sizes of receptive field. The promising results demonstrate the ability of TCNs in handling long-distance video sequence. Moreover, Funke et al. [6] introduce a 3D convolutional neural network (CNN) to extract spatial-temporal features from partially sample video snippets. However, due to the huge computational cost, 3D-CNN can only works with clips [25] rather than the whole video.

From another perspective, multi-task learning has been demonstrated as an efficient method for surgical activities understanding [1, 13, 22]. In [1], the authors present a framework to simultaneously recognize the surgical gesture and predict the surgical task progress from kinematic data. The results prove that the multi-task architecture improves the performance of surgical gesture recognition without any additional human annotation. In another study, Wang et al. [22] use 3D-CNN features to predict the skill score and segment surgical, which further demonstrates the benefits of multi-task learning.

**Fig. 1:**
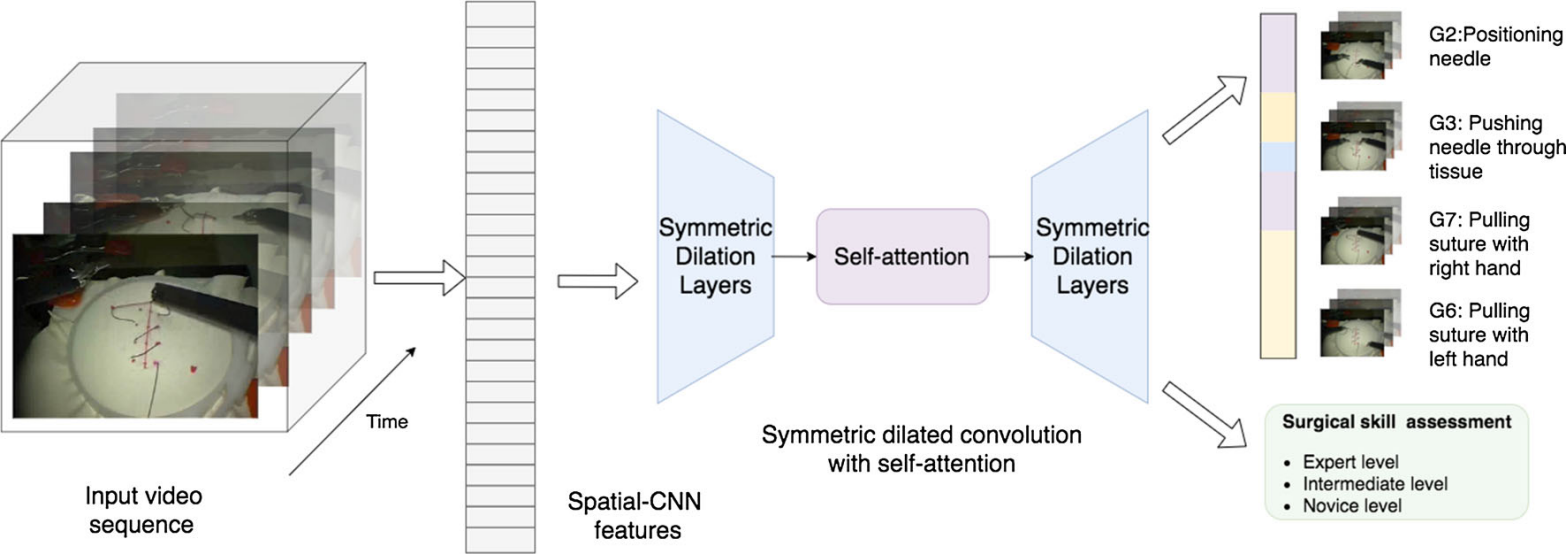
The multi-task architecture for joint surgical gesture segmentation and skill assessment

In this paper, we propose a symmetric dilated convolutional neural network, *SD-Net*, for joint surgical gesture recognition and skill assessment only using RGB videos. Our method is designed on the top of three observations and insights: (1) Surgical video data own high-dimensional and representative features, and it is much easier to access than other data format, for example, kinematic information or motion trajectory; (2) video dynamics is as significant as spatial features in a video sequence; (3) learning with auxiliary task (skill assessment) with global features can benefit gesture segmentation task. Figure 1 shows the overview of our architecture. Rather than using video clips, we take the whole video sequence into consideration. The network is composed of a symmetric dilated encoder–decoder structure to enlarge the receptive field and catch the long-term temporal information and an self-attention module in the middle to build the frame-wise adjacent as well as the global relationships.

Our method has been validated on the suturing task from the JHU-ISI Gesture and Skill Assessment Working Set (JIGSAWS) [8]. This work is an extended version from MICCAI2020 conference paper [24]. The joint learning strategy reveals complementary benefits between gesture recognition and skill assessment, which further improves over our state-of-the-art work in surgical gesture recognition.

**Fig. 2:**
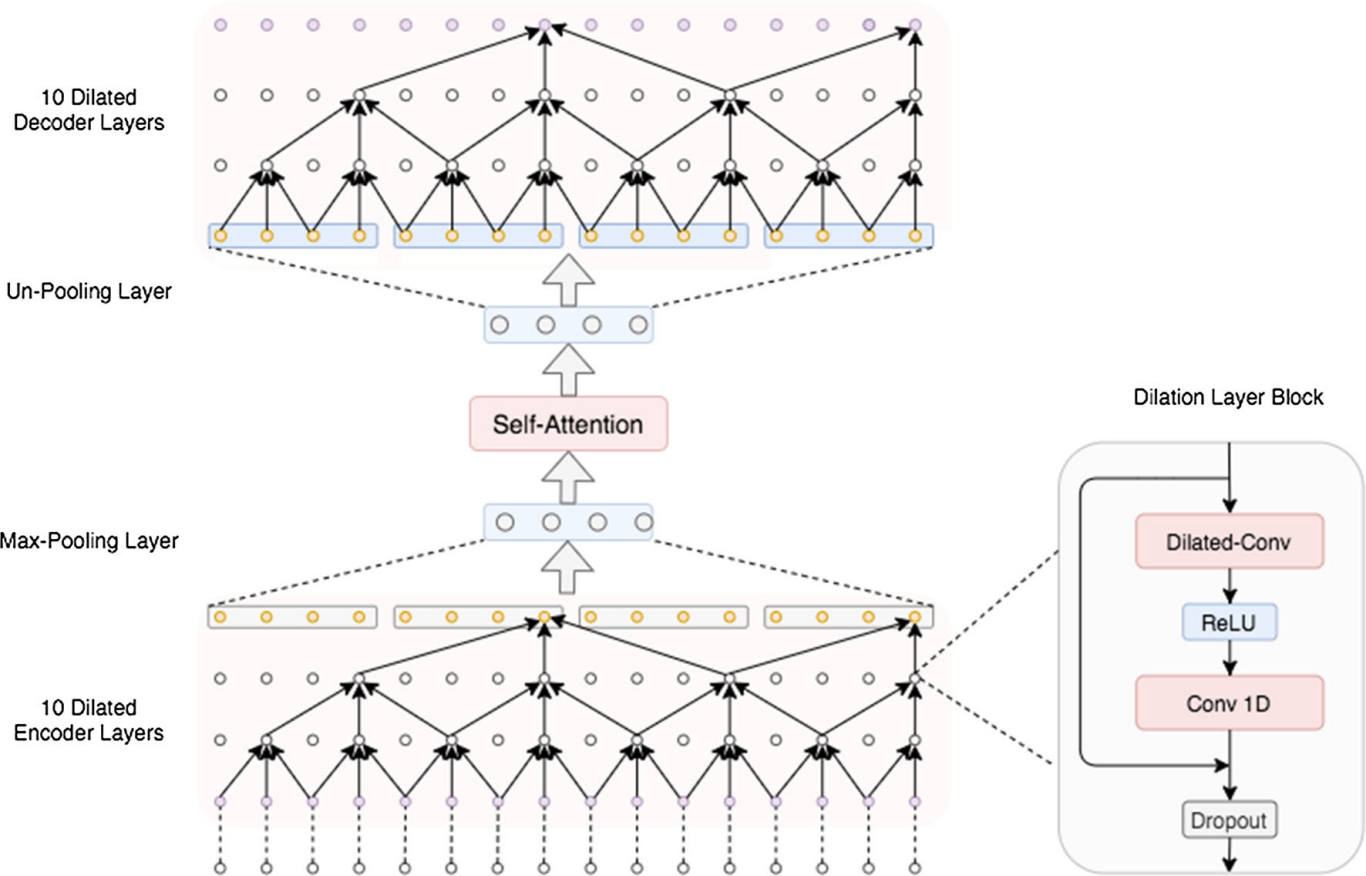
The architecture of SD-Net. The network features a symmetric structure that encode and decode signals with dilated convolutions to aggregate spatial features from hierarchical temporal span. A self-attention module is designed in the middle to bridge the global frame-to-frame adjacency across the full temporal domain. Each node represents a feature vector computed from previous nodes

## Methods

In this section, we introduce the structure design of SD-Net, which consists of three sub-components: (1) temporal dilated convolutional encoder-decoder for capturing multi-scale video dynamics (see Sect. Temporal dilated sonvolutional encoder-decoder); (2) embedded self-attention for connecting and representing every frame in the same sequence (see Sect. Non-local sequence representation by self-attention); and (3) multi-task learning for surgical gesture recognition and skill assessment

### Temporal dilated convolutional encoder-decoder

The whole encoder–decoder structure is shown in Fig. 2. Before our method, we first obtain the frame-wise feature vectors from [11, 12] as the input. Each frame is represented by a 128-dim vector (i.e., bottom nodes in Fig. 2). Our encoder begins with a $1\times 1$ convolution layer with a kernel size of 128 to map the input features into *f*-dim (*f*=128). Then, it is followed by *L* layers of temporal dilated convolutions. The temporal dilated convolution layer is featured with 1-D fully convolutions with a defined sliding gap across temporal domain (i.e., 1-D temporal dilation). The dilation rate $s_{l}$ at the *l*-th layer is set to $s_{l} = 2^l, l=0,1,...,L-1$. *L*=10. With the layer number increasing, the size of receptive fields grows exponentially that enables to capture long-range temporal clues with less parameters. For each dilation layer, we use acausal mode and set the kernel size at 3 following the details in [4] and padding size at 1 to keep the output size consistent with the input. A dilation layer can be formulated as follows:

1 $$\begin{aligned} \hat{E_l}= & {} \mathrm {ReLU} (W_1 * E_{l-1} + b_{1}) \end{aligned}$$

2 $$\begin{aligned} E_l= & {} E_{l-1} + W_2 * \hat{E_l} + b_{2} \end{aligned}$$

where $E_l$ is the output of the *l*-th dilation layer, $W_1 \in {\mathbf {R}}^{f\times f\times 3}$ represents the weights of dilated convolutions with *f* convolutional kernels, $W_2 \in {\mathbf {R}}^{f\times f\times 1}$ denotes the weights of a $1\times 1$ convolution and $b_1, b_2 \in {\mathbf {R}}^f$ are corresponding bias vectors. Every dialed layer is followed by a non-linear activation ReLU and then a residual connection between the input and the output from the $1\times 1$ convolution. Furthermore, we set the dilation rates $s_l$ to $s_{l} = 2^l, l=0,1,...,9$ such that the size of receptive field *R* grows exponentially, $R(l) = 2^{l+1} - 1$, to capture the long term dependencies. Afterward, we also deploy a max-pooling layer with a kernel size of $4\times 1$ as the final layer of our encoder behind the dilation blocks, which presents promising effects on alleviating the over-segmentation issues (see our ablation study).

Symmetrically, we design a similar structure for the dilated decoder. The only difference is that the max-pooling layer has been replaced with a $1\times 4$ unpooling layer. The encoder–decoder architecture is designed in this way to hierarchically accumulate the spatial features from different temporal spans with memory-efficient dilated convolutions.

**Fig. 3:**
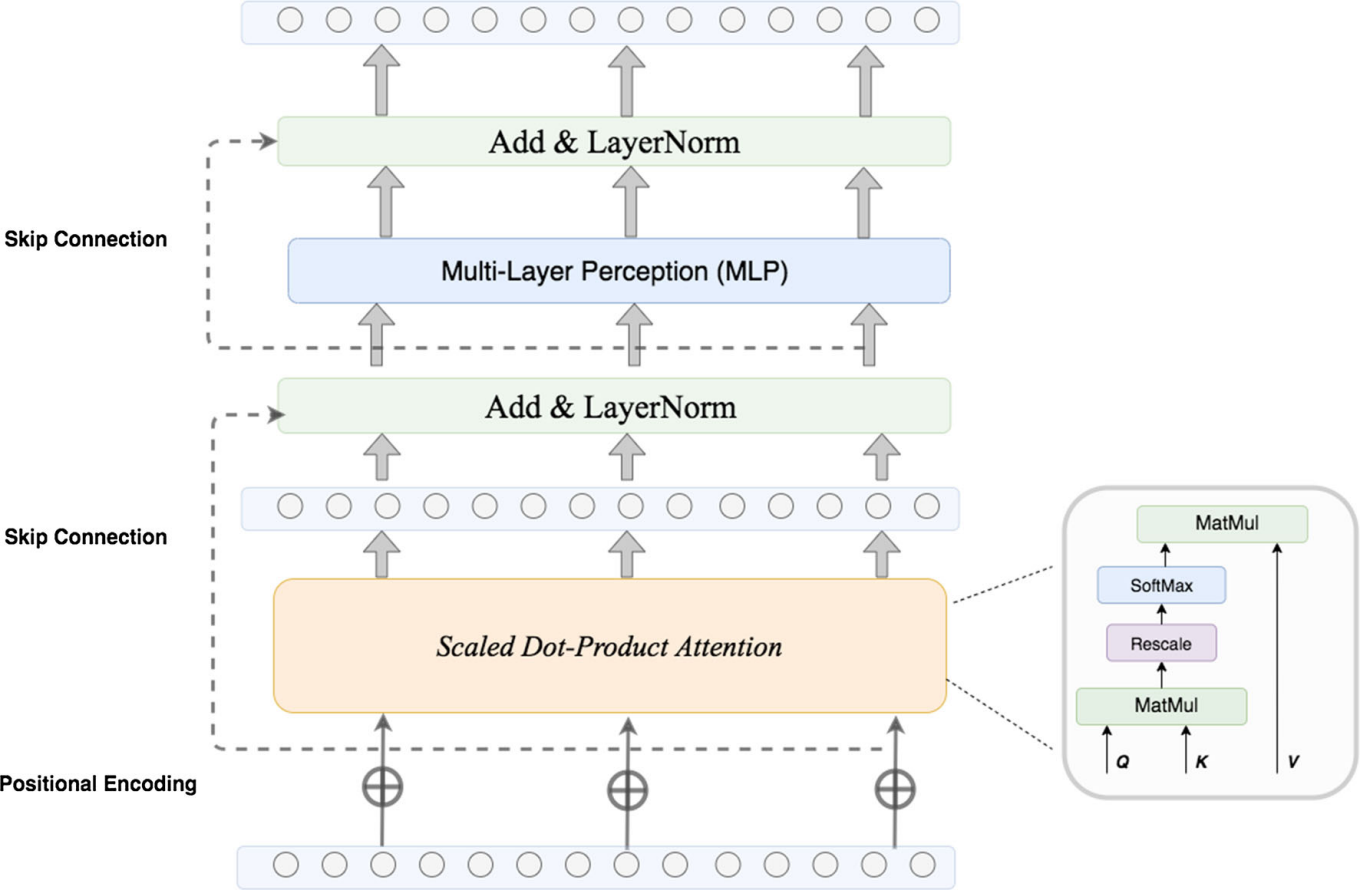
Self-attention module in SD-Net

### Non-local sequence representation by self-attention

Previous works [4, 12] have shown the robustness of TCNs in handing long sequence in variable length. However, these methods are designed with a local manner that employs *relational features* among neighboring frames, thus limit the ability of capturing global information. Self-attention is an attention mechanism to represent an input sequence itself by building one-to-all relationships for all frames. This idea is originally from machine translation [21] and has been widely used in object segmentation [9, 23], image captioning [2], etc. Inspired by this non-local attention mechanism, we embeds an self-attention block (see Fig. 3) between the encoder and decoder to extract frame-to-frame global dependencies. The essential idea behind self-attention is *Scaled Dot-Product Attention*, which is computed as:

3 $$\begin{aligned} \mathrm {Attention} (Q,K,V) = \mathrm {Softmax} \left( \frac{QK^T}{\sqrt{d_k}}\right) V \end{aligned}$$

where *Q* stands for the queries packed as a matrix, *K* and *V* are *Key-Value* pairs and $d_k$ is the dimension of queries and keys. The input *Queries*, *Keys* and *Values* of self-attention are the same, that is the output hidden state from encoder pooling layer. After a positional encoding layer [21] to keep the sequence order, the dot product is calculated between queries *Q* and keys *K* to find the similarity score between every single frame and all other frames. The result then is divided by $\sqrt{d_k}$ to prevent exploding gradient during back-propagation. Softmax function is applied here to normalize the scores. Finally, we multiply each value vector in *V* by the weighted similarity score and sum them up. The output from the scaled dot-product attention layer is then fed into a fully connected network. There are two residual connections followed by layer normalization associating with the self-attention and fully connected layer (see Fig. 3), respectively.

### Joint surgical gesture recognition and skill assessment

For a video sequence $v \in V$ with length *T*: $v_{1:T} = (v_1,...,v_T)$, we aim to assign the gesture label $g \in {\mathbf {G}}$ to each frame: $g_{1:T}=(g_1,...,g_T)$ and the skill level label $y \in {\mathbf {Y}}$ to the whole video *V*, where ${\mathbf {G}}$ has 10 categories and ${\mathbf {Y}}$ has 3 categories. Suppose $D_L$ is the decoder output from the last dilation layer. Then we have:

4 $$\begin{aligned} \begin{aligned} G_t&= \mathrm {Softmax}(W_{3} * D_{L,t} + b_{3})\\ Y&= \mathrm {Softmax}(W_{4} * D_{L} + b_{4}) \end{aligned} \end{aligned}$$

where $W_{3} \in {\mathbf {R}}^{f\times 10}$ and $W_{4} \in {\mathbf {R}}^{f\times 3}$ are network trained weights. Their corresponding bias vectors are represented as $b_{3} \in {\mathbf {R}}^{10}$ and $b_{4} \in {\mathbf {R}}^{3}$. We calculate the categorical cross-entropy loss for both gesture segmentation and skill assessment task. The multi-task loss *L* is the weighted combination of two tasks such that ${\mathcal {L}} = \alpha {\mathcal {L}}_{gesture} + \beta {\mathcal {L}}_{skill}$. Since skill assessment is the auxiliary task for gesture recognition, we set $\alpha = 0.9$ and $\beta = 0.1$.

## Results and evaluation

### Experimental details

**Dataset**: We validate our approach on suturing task from JIGSAWS dataset [8]. The dataset is captured using da Vinci robotic surgical system from eight surgeons with different levels of skill, that is *expert, intermediate and novice*. They perform five repetitions for three elementary surgical tasks on a bench-top model. An experienced surgeon manually annotates 10 fine-grained surgical gestures from the videos such as *pulling suture with left hand* and *dropping suture at end and moving to end points*. In addition, authors of JIGSAWS dataset define two cross-validation schemes: *leave-one-user-out (LOUO)* and *leave-one-supertrial-out (LOSO)*. In each fold, LOUO left all the trails from the *i*-th surgeon (out of eight surgeons) for testing and the rest for training. It is efficient to verify whether a model works for an unseen subject. We use LOUO for gesture recognition evaluation, following the settings in [6, 22]. While LOSO scheme leaves the *i*-th trial (out of five trials) from all the eight surgeons for testing and the rest for training. LOSO scheme is applied for validating the performance of skill assessment task following the previous works [5, 7].

**Evaluation Metrics:** For surgical gesture recognition evaluation, we evaluate our method on both frame level and segmental level, where the *frame-wise accuracy* *edit score* and *segmented F1 score* are, respectively, used as the evaluation metrics. Frame-wise accuracy calculates the percentage of correctly classified frames. However, frame-wise accuracy is insensitive to over-segmentation error. For this reason, we use edit score (the normalized Levenshtein distance) and segmented F1 score (the harmonic mean of precision and recall) to measure the coherence of gesture. For the skill assessment task, we calculate its averaged accuracy over five cross validation runs.

**Hyper-parameters and Training Details:** We implement our SD-Net using Pytorch and train the model on a NVIDIA GeForce GTX 1080 graphics card. We adopt spatial CNN features with 128-dim at 10 FPS from [12]. For the symmetric dilated encoder and decoder, we set the layer number *L* to 10 (see the supplementary material from [24] for detailed ablation experiment) and the convolutional channel *f* to 128 with the kernel size at 3. For the self-attention block, we set the dimension of queries *Q* and keys *K* to 16 and the hidden size of fully connected layer to 512. As for the multi-task loss function, we set the action recognition weight $\alpha$ to 0.9 and skill assessment weight $\beta$ to 0.1 due to their best performance on cross-validation. The network is trained with 30 epochs using Adam optimizer and the learning rate is set to 0.01.

### Experimental results

We compare the performance of SD-Net with other state-of-the-art methods under LOUO validation scheme (see Table 1 for the results), including one kinematic data-based reinforcement learning approach [14] and four video-based approaches [6, 12, 18, 22]. It can be observed that **Bi-LSTM** method achieves the lower performance than other approaches, which implies the limited ability of RNN models in handling long sequences. **RL** network reaches a relatively high segmental-level accuracy (edit score at 87.96 and F1 score at 92.0) but the low frame-wise accuracy is only at 81.43%. It indicates that the learned strategy is not able to catch frame-to-frame similarity in global. The latest **C3D-MTL-VF** model treats surgical gesture recognition as an auxiliary task of skill score prediction by using the multi-stage temporal convolutional network proposed in [4]. The relatively low frame-wise (82.0%) further demonstrates that TCNs focus on the local neighbor rather than the global dependencies, while our SD-Net network with multi-task learning outperforms the state-of-the-arts on all three evaluation metrics. The promising results prove the robustness of our approach in both frame-level and the segmental-level gesture recognition.

In regard to the evaluation of surgical skill assessment task, we follow the LOSO fivefold cross-validation scheme in consistency with other state of the arts [5, 7, 22]. (The reason why we do not use LOUO scheme to evaluate the performance of skill assessment is discussed in Sect. LOUO skill assessment.) Comparing with other prior studies [5, 7, 22], we keep the 100% accuracy score for the suturing task.

**Table 1:** Performance comparison of surgical gesture recognition on suturing task averaged over eight cross-validation runs under LOUO scheme. Acc., Edit and F1@{10, 25, 50} represent the frame-wise accuracy, edit distance and F1 score in different thresholds

Suturing (LOUO)	Acc.	Edit	F1@10	F1@25	F1@50
Bi-LSTM [18]	77.4	66.8	77.8	–	–
ED-TCN [12]	80.8	84.7	89.2	-	–
RL [14]	81.43	87.96	92.0	90.5	82.2
3D-CNN [6]	84.3	80.0	87.0	–	–
C3D-MTL-VF [22]	82.0	86.6	90.6	89.1	80.3
SD-Net [24]	90.1	89.9	92.5	92.0	88.2
SD-Net (w. multi-task)	**90.5**	**90.6**	**93.5**	**92.8**	**90.5**

Bold values indicate the comparing with all the listed methods, which one reaches the highest evaluation score of that column (evaluation metric)

**Fig. 4:**
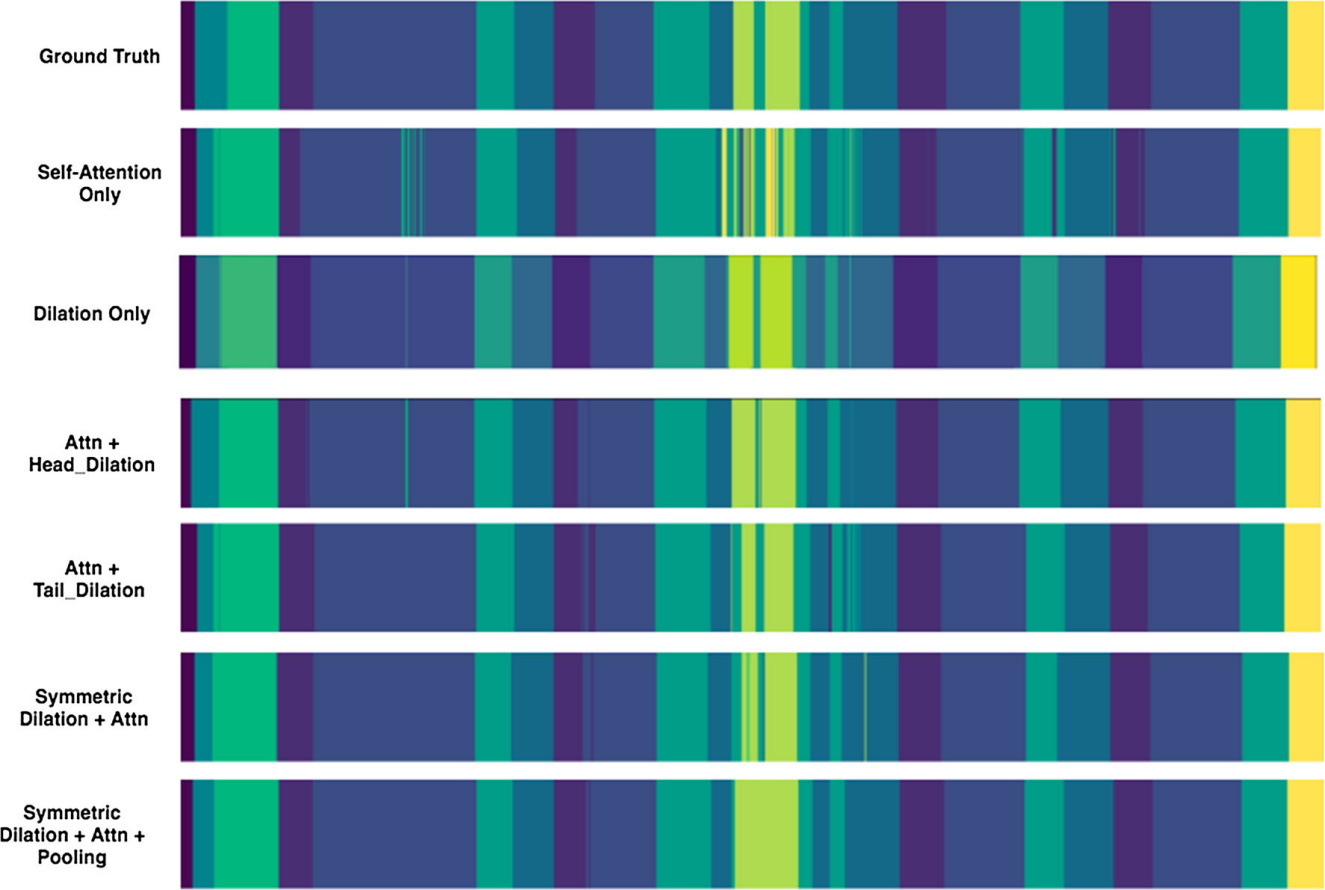
Visualization results from ablation study

## Discussion

### Ablation study

In order to explore the effectiveness of each single design in our method, we decompose our network into six configurations as follows:

***C0:*** self-attention only (baseline)

***C1:*** dilation layers only (baseline)

***C2:*** dilated convolutional encoder + self-attention

***C3:*** self-attention + dilated convolutional decoder

***C4:*** symmetric dilated convolution + self-attention

***C5:*** symmetric dilated convolution + self-attention + pooling (SD-Net)

***C6:*** SD-Net + skill assessment branch

Every configuration runs under LOUO scheme with eight-fold cross validation. The results are presented in Table 2 and Fig. 4, from which we observe that:

***C0 v.s.*** ***C2*** and ***C3***: The model with only self-attention block reaches high frame-wise accuracy at 87.8% but very low Edit and F1 score in different threshold. When we add temporal dilated convolution module, the segmental-level performance improves around 30% in each metric. It indicates that dilated temporal convolution presents promising ability in capturing long-term information.

***C1 v.s.*** ***C2*** and ***C3***: Self-attention module is able to catch non-local frame-wise information. Adding self-attention on top of the dilation layers improves the performance over all evaluation metrics.

***C2*** and ***C3*** ***v.s. C4***: The whole symmetric encoder–decoder structure further improves the segmental-level performance comparing the single sided structure.

***C4*** ***v.s.*** ***C5***: From Fig. 4 and Table 2, we observe that the pooling strategy alleviates the over-segmentation problem that further improves the Edit and F1 scores in different thresholds.

***C5*** ***v.s.*** ***C6***: Learning with auxiliary task benefits the segmentation of surgical gesture without decreasing the performance of surgical skill assessment.

**Table 2:** Ablation experiments of surgical gesture recognition on suturing task averaged over eight cross-validation runs under LOUO scheme. Acc., Edit and F1@{10, 25, 50}, represent the frame-wise accuracy, edit distance and F1 score in different thresholds

Suturing (LOUO)	Acc.	Edit	F1@10	F1@25	F1@50
Self-attn only	87.8	44.0	54.8	53.5	49.0
Dilation only	90.1	76.8	81.9	81.5	78.5
Encoder dilation+ attn	**90.8**	76.9	82.5	81.8	79.3
Decoder dilation +attn	90.5	77.9	83.4	83.4	79.7
Symmetric dilation + attn	90.7	83.7	87.7	86.9	83.6
Symmetric dilation + attn + pooling	90.1	89.9	92.5	92.0	88.2
SD-Net (w. multi-task)	90.5	**90.6**	**93.5**	**92.8**	**90.5**

Bold values indicate the comparing with all the listed methods, which one reaches the highest evaluation score of that column (evaluation metric)

### LOUO skill assessment

Although LOUO validation is efficient to test whether a model works for a new subject, data from JIGSAWS are insufficient to support such a validation. One reason is because of the limited data size and imbalanced label. JIGSAWS only contains 8 subjects in total, including only two experts and two intermediate-level surgeons. In one validation round, if we left one expert for testing and the other subjects for training, then we only have one expert in the training set. This is also the case for intermediate-level surgeons. On the other hand, the official dataset defined the expertise level of a surgeon in accordance with the robotic surgical experience by hours: experts have more than 100 hours experience, intermediate subjects have 10 to 100 hours experience, and novices have less than 10 hours experience. Nevertheless, some of the intermediate surgeons get higher score than experts for their performance in skill annotation.

**Fig. 5:**
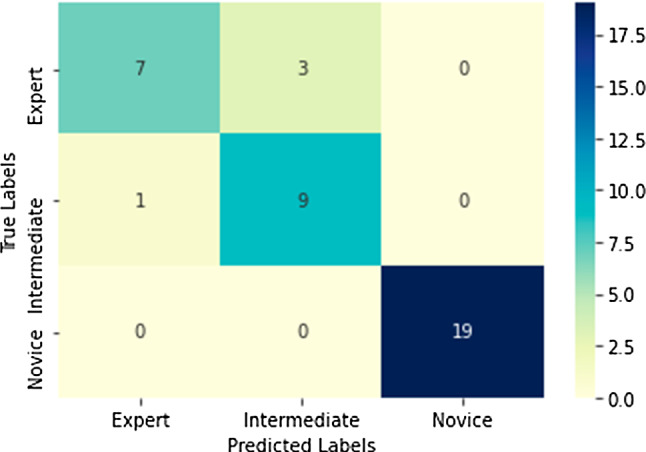
Confusion matrix of the suturing task, obtained from LOUO evaluation

We perform the LOUO fivefold cross-validation for skill assessment and get the average accuracy at 89.7%. We further visualize the accumulative confusion matrix for the result as shown in Fig. 5. It can be seen that we correctly classify the novice sample, while there are some mis-classifications between expert and intermediate surgeons (as mentioned above). In the future work, the current dataset needs to be extended with more subjects in different skill levels.

## Conclusion

In this paper, we present the SD-Net for joint surgical gesture recognition and skill assessment from untrimmed surgical videos. This network consists of a symmetric dilated encoder–decoder structure to learn long-term video dynamics and a self-attention network in the middle to extract frame-to-frame relationship to capture global dependencies. The experiments show that our joint learning approach presents better performance than learning with single tasks. It significantly outperforms the state of the arts on surgical gesture recognition task while keeping the high performance on skill assessment task. It implies that each surgical video understanding task has a complementary effect on others that reveals the future direction of building a context-aware system with holistic surgical process understanding.
